# Association Between China’s DRG Reform and Value-Based Healthcare Orientation: Evidence From Inpatients With Acute Myocardial Infarction and Cancer in Tertiary Hospitals in Western China

**DOI:** 10.34172/ijhpm.9669

**Published:** 2026-07-14

**Authors:** Haoran Li, Fukun Zhu, Tao Xie, Ning Zhang, Austen El-Osta, Zhonghan Wang, Ying Mao

**Affiliations:** ^1^School of Public Policy and Administration, Xi’an Jiaotong University, Xi’an, China.; ^2^School of Public Health, Imperial College London, London, UK.; ^3^School of Political Science and Public Administration, Wuhan University, Wuhan, China.; ^4^Vanke School of Public Health, Tsinghua University, Beijing, China.

**Keywords:** Value-Based Healthcare, Diagnosis-Related Groups, Interrupted Time Series, Acute Myocardial Infarction, Cancer, Western China

## Abstract

**Background:**

Implementing diagnosis-related groups (DRGs) reform under the guidance of value-based healthcare (VBH) is of great significance for China to curb the rapid rise of medical costs and promote the allocation and utilization of medical resources. However, current research evaluating the effectiveness of China’s DRGs reform lacks a value-based oriented approach to healthcare and has not yet explored whether different types of diseases and population characteristics will affect policy implementation.

**Methods:**

A total of 16 564 acute myocardial infarction (AMI) and 74 356 cancer inpatients were included in this single-center study during 2018-2022. Interrupted time series models were used to estimate post-reform level and slope changes. Exploratory subgroup analyses were conducted by gender, age, and disease type. Hospital-level outcomes included risk-adjusted success per cost (SPC), cost per success (CPS), and net monetary benefit (NMB). Individual-level outcomes included total hospitalization cost, length of stay, mortality, successful outcomes, and 30-day readmission.

**Results:**

DRG reform was associated with changes in VBH indicators, with AMI mainly showing slope changes and cancer mainly showing level changes. However, individual-level analyses showed that some clinical outcomes did not improve consistently, which differed from the hospital-level results.

**Conclusion:**

The findings suggest that DRG reform was associated with changes in selected hospital-level VBH indicators, while some individual-level clinical outcomes did not show consistent improvement. Future payment reform should combine disease-specific clinical pathways, stronger risk adjustment, and routine monitoring of patient-level quality outcomes, especially for acute and high-risk conditions.

## Background

**Key Messages**

**Implications for policy makers**

- Our findings suggest that China’s diagnosis-related group (DRG) reform was associated with changes in selected hospital-level value-based healthcare (VBH) indicators, while some individual-level clinical outcomes did not show consistent improvement.
- The observed post-reform changes differed across diseases: Acute myocardial infarction (AMI) was mainly characterized by gradual slope changes, whereas cancer was mainly characterized by immediate level changes. Payment design should therefore consider disease-specific clinical pathways and risk profiles rather than relying on a single national template.
- Exploratory analyses suggested age-related differences among AMI patients, but these subgroup findings should be interpreted cautiously because formal interaction testing was not conducted. Policy-makers should consider protective adjustments for high-risk or clinically complex groups to maintain equity.
- To deepen reform, integrating payment methods with clinical pathway development, establishing risk-adjustment and protection mechanisms for high-risk patients, and monitoring individual-level health outcomes will be essential for building a sustainable VBH-oriented payment system.

**Implications for the public**

This study shows that China’s diagnosis-related group (DRG) payment reform was associated with changes in selected value-based healthcare (VBH) indicators. Some hospital-level indicators changed in a direction consistent with greater cost-outcome efficiency, but several individual-level outcomes did not improve in parallel. Exploratory analyses suggested more pronounced changes among older acute myocardial infarction (AMI) patients, although these findings require further validation. For the public, this means that payment reform may help hospitals use resources more efficiently, but cost control should not be the only goal. Ongoing monitoring of mortality, readmissions, care quality, and high-risk groups is needed.

Value-based healthcare (VBH) was first proposed by Michael Porter in 2006. It refers to the organizational and management concept of healthcare services that is oriented towards creating health value. It emphasizes a people-centred approach that aims to maximize health outcomes at a lower cost.^1,2^ The concept of VBH aims to improve resource efficiency, enhance health outcomes, and elevate population health by shifting health systems from volume-based care to a patient-centered, outcome-oriented model, and has become a key direction for global health system reform.^3,4^ In 2016, the concept of VBH was introduced to China and reinforced by a strategy supported by the World Bank and the World Health Organization (WHO).^5^ Subsequently, the concept was incorporated into various documents for deepening the healthcare system reform in China, crystallising into a shared goal for advancing high-quality healthcare service and medical security.

 Over the past decade, with the expansion of medical insurance coverage, changes in the disease spectrum and increased demand for health among the people, the imbalance between medical costs and benefits has become a serious challenge threatening China’s medical and health area. The long-term implementation of fee-for-service payment has been regarded as one of the main reasons for excessive medical treatment and the unreasonable increase in medical expenses.^6^ To address this, in 2018, China proposed to reform the medical insurance payment method, and in 2019, it officially launched policy pilot programs in 30 cities. This was followed in 2021 by the introduction of a three-year action plan for the reform of diagnosis-related groups (DRGs) and diagnosis-intervention packet payment method, clearly stating that by 2025, the payment method based on DRG and diagnosis-intervention packet should cover all suitable medical institutions that can provide inpatient services and achieve full coverage of all diseases.^7^ The shared goal of the reform of medical insurance payment methods was to control the unreasonable growth of medical expenses, and to serve as a core economic lever to promote VBH.^8^ Currently, China’s payment reform has improved service efficiency and cost control, but it has yet to establish a clear value-oriented framework, as current mechanisms focus more on regulating provider behavior and expenditures than on patient health outcomes.^9^

 Most studies suggest that DRG payment reduces medical costs and length of stay,^8,10^ but some evidence indicates that while drug costs decline, treatment fees and patients’ out-of-pocket payments may increase.^11^ At the same time, studies highlighted that after the DRG reform, hospitals tend to admit patients with lower costs or lower risks to optimize the budget structure and income while also excluding complex cases which may negatively impact equity in access and quality of medical services, highlighting gaps in measuring the impact of DRG on health outcomes.^9,12^ Some Chinese scholars have attempted to evaluate changes associated with China’s DRG reform from the perspective of VBH and incorporate treatment costs such as average per capita expenses and medical service quality indicators such as case mix index values to assess the reform effect.^13,14^ However, these studies relied on institutional-level data and did not truly adopt patients’ health outcome indicators, meaning that they could not reflect the people-oriented concept in VBH.^15^ Previous studies also could not determine whether the reform effect of DRG varies due to demographic differences, whereas related studies mostly focused on single disease scenarios such as myocardial infarction and cancers, or single department scenarios such as cardiology and orthopaedics. These studies, whilst helpful, lacked comparative analyses of DRG reform effects based on different disease types, resulting in certain limitations in external promotion and generalizability of the research conclusions.^15-17^ For these reasons, understanding the contribution of DRG payment reform to VBH is crucial. It necessitates research to determine whether the current reform is helping the healthcare system become more efficient, fair and of higher quality, and whether it supports China’s continued advancement of its VBH strategy.

 To address these gaps, the present study utilizes longitudinal data from a large hospital in western China to examine post-reform changes associated with DRG reform in the health outcomes and treatment costs of inpatients with two diseases: acute myocardial infarction (AMI) and cancer. The primary objective of this study was to assess the association between China’s DRG payment reform and VBH performance, based on data from inpatients with AMI and cancer. Specifically, our objectives are: (*i*) to evaluate changes in hospital-level VBH indicators and individual-level cost and clinical outcomes after DRG reform, (*ii*) to examine whether post-reform change patterns differed between AMI and cancer treatments, and (*iii*) to explore whether these changes varied by gender and age.

## Methods

###  Setting and Data Sources

 The data used in this study was sourced from a large public hospital in a municipality in the west of China designated as a national pilot site for DRG reform. The hospital was one of the first in the region to implement DRG in 2021, operating under a pilot programme for one year before full formal adoption in 2022. Through an interview with the officer in charge of the hospital’s medical insurance management department, we learned that in 2021, the hospital only conducted a trial run of DRG during the pilot program and did not use it for formal settlement. Therefore, we also included the data of 2021 (without using DRG for settlement) as the research period. The study therefore draws on five complete pre- and post-reform years of data (2018, 2019, 2020, 2021, and 2022), providing a total observation period of 60 months.

 This study focused on two major disease scenarios for inpatient care: AMI and cancer (specifically malignant tumors of the breast, prostate, lung, and intestine). AMI has the characteristics of sudden onset and severe life-threatening nature, requiring prompt provision of inpatient rescue and treatment. Whereas the hospitalization duration for AMIs is relatively short, the treatment cost is relatively high although clear treatment outcomes are produced within a short period. Conversely, cancer often requires prolonged treatment cycles and generating high care costs. More severe cases necessitate extended hospital stays and coordinated multidisciplinary management, making cancer a key indicator of a health system’s capacity to manage complex and major diseases. The two conditions both constitute significant challenges for health management in China and are widely recognised as typical, representative high-burden conditions.

 The raw data used in this study were extracted from the hospital information system (HIS) and the medical record homepage database, with the assistance of the hospital information department, the medical insurance administration office, and clinicians from the relevant departments. The inclusion criteria for eligible patients were jointly determined by the medical insurance office, the relevant clinical departments, and the research team. The hospital information department then extracted the data based on disease codes and admission identifiers, and all records were de-identified during extraction to ensure data privacy and confidentiality. As the data were obtained directly from the HIS, they constitute standardized structured data. The definitions, values, and measurement of all variables followed the unified HIS and medical record homepage specifications of the municipality, and the research team did not independently define or subjectively measure any variables. The data are stored and managed on secure institutional servers, and only de-identified data were accessible to the research team. Due to institutional data governance and patient privacy regulations, the raw data are not publicly available but may be provided for academic purposes upon reasonable request and with approval from the hospital administration.

 Data preparation focused on the consistency and plausibility of the standardized administrative records. The extracted data were checked for duplicate admission identifiers, invalid disease codes, implausible admission and discharge dates, and missing key variables required to define the study population, time point, cost, and clinical outcomes. Disease and procedure coding were harmonized according to the municipal HIS and medical-record homepage specifications. Because the data were administrative rather than clinically adjudicated research records, independent validation of every diagnosis and outcome was not possible.

###  Study Design

####  Study Population

 A total of 16 564 AMI and 74 356 cancer inpatients were included in this study. The primary diagnosis of the disease was provided by the hospital system and linked with medical record front sheet containing sociodemographic characteristics, hospitalization details and clinical outcomes. Descriptive characteristics are reported in Table 1.

**Table 1:** Characteristics of Inpatients Diagnosed With Acute Myocardial Infarction and Cancers

**Characteristics**	**AMI (N = 16** **564)**		**Cancer (N = 74** **356)**	
	**N/Mean**	**%**	**N/Mean**	**%**
Gender				
Male	13 576	81.96	34 346	46.19
Female	2988	18.04	40 010	53.81
Age	62.06	-	59.02	-
<45	1202	7.26	9585	12.89
45-59	5497	33.19	26 770	36.00
≥60	9865	59.56	38 001	51.11
Marriage				
Widowed	164	0.99	455	0.61
Married	16 217	97.91	72 703	97.78
Single	120	0.72	793	1.07
Divorced	60	0.36	378	0.51
Other	3	0.02	27	0.04
Nation				
Han	16 481	99.50	74 034	99.57
Other	83	0.50	322	0.43
LHS	5.46	-	5.56	-
Level				
1	60	0.36	589	0.79
2	71	0.43	3063	4.12
3	381	2.30	3911	5.26
4	398	2.40	11 673	15.70
5	14 761	89.11	10 539	14.17
6	893	5.39	44 581	59.96
Outcomes				
1	1033	6.24	28 587	38.45
2	14 913	90.03	40 633	54.65
3	33	0.20	263	0.35
4	157	0.95	121	0.16
5	428	2.58	4752	6.39
Total fee	124 589.40		25 634.46	
The number of doctors	1.18		1.11	
The number of assistants	1.09		0.60	

Abbreviations: AMI, acute myocardial infarction; LHS, length of hospital stay.

####  Outcomes

 This study recorded the total fee of a single hospitalization and the health outcomes at the patient level. The health outcomes were classified into five categories: cured, improved, not cured, death, and other (such as transfer to another hospital, indicating that the patient was not cured during this hospitalization). Based on this, we introduced two types of outcome indicators: cost indicators and clinical outcome indicators at individual-level, and VBH indicators at the hospital-level. Among them, the cost indicators at individual-level were the total cost of hospitalization. The total cost of hospitalization refers to the full expenditure associated with a single inpatient episode at the individual patient level. The clinical outcome indicators at individual-level included the length of hospital stay (LHS) at the patient level, mortality, successful outcome rates (cured and improved) constructed from hospitalization outcomes and 30-day readmission.

 Subsequently, we constructed regression models for each patient, separately for cost indicators and clinical outcome indicators, incorporating case-mix variables such as age, gender and severity of other diseases. For continuous outcomes, we used a log-linked Gamma generalized linear model (GLM), and for binary outcomes, we used a logistic regression model. This allowed us to estimate the expected outcome for each patient given their specific risk characteristics. Individual-level actual values and expected values by month were then aggregated to obtain the observed and expected average values for each month. We constructed risk-adjusted monthly indicators based on the average outcome of the total sample, as shown in formula 1. This enabled us to compare the changes in outcome levels before and after the reform and at different time points, while eliminating the influence of differences in age, gender, and disease severity. The risk-adjustment procedure could be interpreted as administrative case-mix adjustment rather than complete clinical severity adjustment. It used observable demographic, socioeconomic, diagnostic, and treatment-intensity variables available in the HIS and medical-record homepage data.


(1) $$RA_{t}=\frac{1}{N}\sum_{i=1}^{N}Y_{i}+(Observed_{t}-Expected_{t})$$

After constructing the risk-adjusted clinical outcome indicators at the hospital-level, we calculated three types of VBH metrics: Cost per success (CPS), success per cost (SPC), and net monetary benefit (NMB). CPS reflected the average cost required to achieve one successful outcome (total cost/risk-adjusted total successful outcome cases), expressed in 10 000 CNY.^18^ SPC assessed the efficiency of medical services from the perspective of “benefit/cost,” measured in the number of successful outcomes per 10 000 CNY of cost (risk-adjusted total successful cases/total cost).^19,20^ To comprehensively measure cost and successful outcomes, this study adopted NMB.^21^ NMB is commonly used in health economic evaluation to express health benefits and costs on a common monetary scale and to support value-based comparisons across interventions. In this study, because there is no unified willingness-to-pay threshold for one successful inpatient outcome in China, the monetary value of achieving one successful outcome was estimated using the adjusted median CPS in the last 12 months before payment reform. This value was multiplied by the risk-adjusted number of patients who achieved successful outcomes in each month, and the total medical expenditure in the same month was then subtracted to obtain NMB. Given the absence of a unified external willingness-to-pay threshold for successful inpatient outcomes, NMB was treated as an exploratory hospital-level value indicator in this study. SPC, CPS, and NMB were selected because they capture complementary dimensions of healthcare value, including cost efficiency, service output efficiency, and net value, within a unified analytical framework. SPC reflects the amount of service output generated per unit of resource, CPS represents the cost required to achieve a given health outcome, and NMB integrates costs and outcomes into a single value measure. Together, these indicators operationalize the cost–outcome relationship at the core of VBH and provide a multidimensional assessment of institutional performance under DRG reform. We conducted sensitivity analyses by redefining the NMB threshold using the 25th percentile and the mean. Because SPC, CPS, and NMB have not been externally validated as VBH measures for Chinese inpatient DRG evaluation, they were used as exploratory hospital-level indicators in this study.

###  Variables

 The covariates in this study included demographic and socioeconomic variables as well as surgery-related variables. Demographic variables included age (age at admission, continuous), gender (male/female), marital status (married, unmarried, divorced, widowed, other, categorical), ethnicity (Han/Minority) and occupation. Surgery-related variables included the main diagnosis disease (categorical), the level of surgery measured on a scale of 1-5 (categorical), and the number of surgeons and assistants involved in the surgery (continuous).

 Surgery level categories were retained according to the medical-record homepage classification (Levels 1-5) and were used as recorded to proxy treatment complexity. Successful outcomes were defined as discharge outcomes recorded as cured or improved.

###  Statistical Analysis

 This study considered each month as a time unit and employed interrupted time series (ITS) regression to assess level and slope changes after DRG reform. Analyses were conducted at two levels: the hospital-level (monthly risk-adjusted indicators) and the individual-level. For hospital-level outcomes, we applied a Prais–Winsten regression with robust standard errors (Formula 2).^22^ Referring to previous studies,^23,24^ at the patient level, we estimated the level change and slope change after DRG reform using individual admission records, while adjusting for patient characteristics. Compared with aggregating patient outcomes into monthly averages, this approach allowed us to retain individual-level information and account for differences in demographic, socioeconomic, and clinical characteristics across patients. For continuous outcomes, we used a GLM with robust standard errors (Formula 3). For binary clinical outcomes, we fitted logistic regression model with the same ITS specification and covariates (Formula 4). All ITS models were constructed to estimate the level (β_1_) and slope (β_2_) changes.^25^ We reported all effect estimates and their 95% confidence intervals (CIs). For the primary outcome, *P* < .05 was considered statistically significant. Stata 15.1was used for statistical analysis in this study.

 Because this was a single-center uncontrolled ITS study, all coefficients were interpreted as temporal changes associated with DRG reform rather than as definitive causal effects. Reporting was guided by Strengthening the Reporting of Observational studies in Epidemiology (STROBE) principles and methodological recommendations for ITS evaluations, including clear specification of the interruption point, pre- and post-reform periods, level and slope terms, concurrent policy controls, autocorrelation diagnostics, seasonality checks, and robustness analyses.


(2) $$Y_{t}=\beta_{0}+\beta_{1}Time_{t}+\beta_{2}Post_{t}+\beta_{3}(Time_{t}-T_{0})Post_{t}+\mu_{t}$$

(3) $$\log(E(Y_{it}))=\beta_{0}+\beta_{1}Time_{t}+\beta_{2}Post_{t}+\beta_{3}(Time_{t}-T_{0})Post_{t}+\beta^{\prime }X_{it}$$

(4) $$\log it(P(Y_{it}=1))=\beta_{0}+\beta_{1}Time_{t}+\beta_{2}Post_{t}+\beta_{3}(Time_{t}-T_{0})Post_{t}+\beta^{\prime }X_{it}$$

###  Robustness Test

 In the robustness analysis, to reduce potential interference from concurrent policies, we incorporated policy variables implemented during the same period into the baseline ITS model. Specifically, lockdown measures, the medical service price adjustment (MSPA), and the centralized drug procurement program (CDPP) were included as covariates. The baseline model was then re-estimated using Prais-Winsten regression to assess whether the post-reform estimates were sensitive to these observed concurrent policies. These analyses were intended to evaluate the consistency of the estimates.

 Meanwhile, we conducted several additional regression models. At the institutional level, we re-estimated the benchmark ITS model using Newey-West regression,^26^ which provides heteroscedasticity- and autocorrelation-consistent standard errors. This approach accounts for potential high-order autocorrelation in the time series, allowing us to examine the consistency of the estimates under an alternative method for handling serial correlation.

 At the individual-level, we conducted additional robustness checks. First, we clustered standard errors by month to account for correlation among individual observations within the same time period. Second, we fitted mixed-effects models,^27^ setting random effects at the individual-level to control the intra-individual correlation arising from repeated observations, thereby testing the stability of the main results. Similarly, we reported all effect estimates and their 95% CIs. For the primary outcome, *P* < .05 was considered statistically significant. Third, to further address temporal structure in the patient-level analyses, we re-estimated the patient-level models by simultaneously including month-of-year fixed effects and clustering standard errors by calendar month. This specification was used to account for potential seasonality and correlation among admissions occurring within the same time period. Given the number of outcomes, robustness checks, and subgroups examined, the subgroup and secondary outcome analyses were treated as exploratory.

 Additionally, to assess potential COVID-19–related disturbances in the pre-intervention period, we conducted additional pre-trend and sensitivity analyses at both the hospital and patient levels. First, using 2018–2021 pre-reform data only, we added a COVID-19 period indicator, defined as January 2020 onward, to the pre-reform trend models. We then re-estimated the main models with adjustment for the COVID-19 period to examine the robustness of the post-reform level and slope estimates.

###  Subgroup Analysis

 After analysing data from all inpatients, we adopted the same analytical steps (hospital-level using Prais-Winsten regression and individual-level using GLM and logistic regression). We conducted exploratory subgroup analyses based on gender and age to examine whether post-reform change patterns varied across patient groups. The gender subgroups included male and female groups, while the age subgroups included those younger than 45 years, those aged 45 to 60 years, and those older than 60 years. Because formal interaction testing was not conducted, these analyses were interpreted as exploratory rather than confirmatory.

## Results

###  Descriptive Characteristics of Inpatients

Table 1 presents descriptive statistics highlighting systematic differences in demographic characteristics, healthcare resource utilization and clinical outcomes between hospitalized patients with AMI, and those with cancer. Male patients predominated in the AMI group (81.96%), whereas the gender distribution was relatively balanced in the cancer group (46.19% male vs. 53.81% female). Regarding age distribution, AMI patients had a higher mean age (62.06 years), with 59.56% aged ≥60. Cancer patients exhibited a slightly lower mean age (59.02 years), and the proportion of younger patients (<45 years) was significantly higher than that in the AMI group (12.89% vs. 7.26%). Most patients in both groups were married (97.91% for AMI vs. 97.78% for cancer). Ethnic composition was highly consistent, with the Han ethnicity comprising over 99.5% in both groups. With respect to healthcare resource utilization, the median total medical cost was substantially lower among cancer patients (CNY 25 634.46) compared to AMI patients (CNY 124 589.40).

 A notable disparity was observed in the distribution of surgical levels: cancer patients underwent high-level surgeries (Levels 1-3) at a higher collective proportion (10.17%), while AMI patients predominantly received lower-level surgeries (Level 5, 89.11%). Additionally, AMI patients also had a greater number of interventionists involved in their treatment. Significant differences were also evident in clinical outcomes. The probabilities of cure and improvement for AMI patients were 6.24% and 90.03% respectively, but much higher for cancer patients (38.45% and 54.65% respectively). Correspondingly, the mortality rate of cancer patients (0.16%) was slightly lower than that of AMI inpatients (0.95%).

###  Impact of Diagnosis-Related Group Reform on Value-Based Performance at the Hospital-Level

 Adjusted changes in value-based performance associated with DRG reform using Prais-Winsten regression are presented in Table 2. For AMI patients, the changes were mainly reflected as a trend change. Following implementation, each additional time unit was associated with an increase of 0.001 in SPC (Coeff. = 0.001; 95% CI: 0.0004 to 0.002), a decrease of 0.162 in CPS (Coeff. = -0.162; 95% CI: -0.256 to -0.068), and an increase of 42.253 in NMB (Coeff. = 42.253; 95% CI: 3.768 to 80.737). These results suggest that changes in AMI hospital-level VBH indicators were mainly observed over time rather than immediately after reform.

**Table 2:** Adjusted Changes in Value-Based Performance Associated With Diagnosis-Related Group Reform Using Prais–Winsten Regression

**Characteristics**	**Change**	**AMI (N = 60)**		**Cancers (N = 60)**	
		**Coeff.**	**95% CI**	**Coeff.**	**95% CI**
SPC	Level change	-0.002	-0.006~0.003	0.100***	0.070~0.131
	Slope change	0.001**	0.0004~0.002	-0.011	-0.035~0.013
CPS	Level change	0.323	-0.442~1.088	-0.362***	-0.546~-0.178
	Slope change	-0.162**	-0.256~0.068	0.035	-0.123~0.193
NMB	Level change	-80.350	-311.579~150.880	407.613*	43.380~771.847
	Slope change	42.253*	3.768~80.737	-10.256	-173.999~153.487

Abbreviations: AMI, acute myocardial infarction; CI, confidence interval; SPC, success per cost; CPS, Cost per success; NMB, net monetary benefit; Coeff., coefficient. Note: * *P* < .05, ** *P* < .01, *** *P* < .001.

 In contrast, the reform effect of DRG for cancer patients mainly manifested as an immediate level change. After the implementation, the SPC of cancer patients immediately increased by 0.100 (Coeff. = 0.100; 95% CI: 0.070 to 0.131), the CPS immediately decreased by 0.362 (Coeff. = -0.362; 95% CI: -0.546 to -0.178), and the NMB immediately increased by 407.613 (Coeff. = 407.613; 95% CI: 43.380 to 771.847). These results suggest that the observed changes were mainly characterized by an immediate level shift. The visualized results are shown in Figure. Table S1 (Supplementary file 1) presents the Durbin–Watson statistics before and after the Prais–Winsten regression, indicating that the residual autocorrelation has been adequately addressed. Model fit statistics and multicollinearity diagnostics for the risk-adjustment and individual-level models are presented in Table S2.

**Figure:**
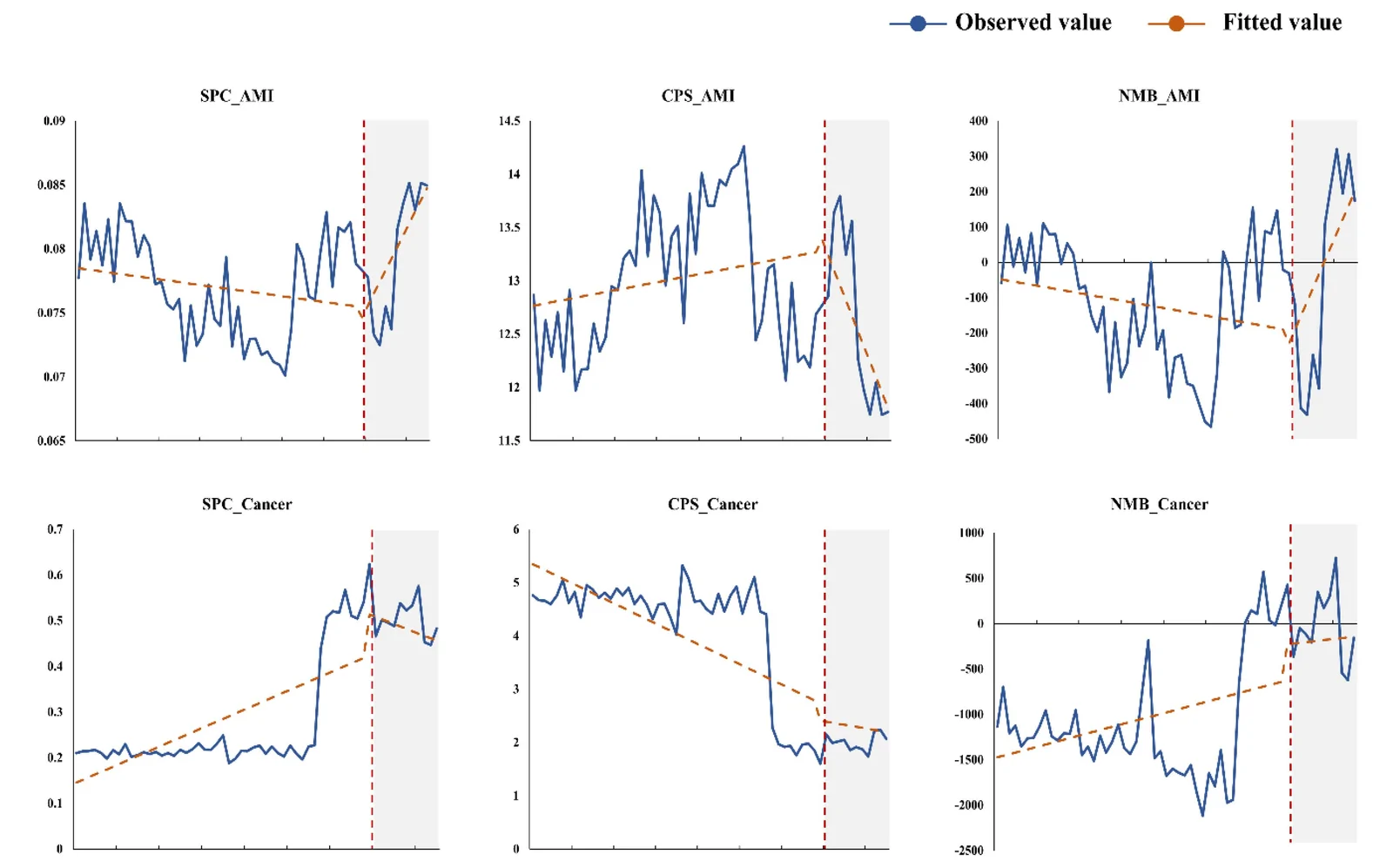
Trends in Risk-Adjusted Hospital-Level Value-Based Healthcare Indicators Among Acute Myocardial Infarction and Cancer Patients Before and After Diagnosis-Related Group Reform. Abbreviations: AMI, acute myocardial infarction; SPC, success per cost; CPS, Cost per success; NMB, net monetary benefit. Notes: The vertical dashed line indicates the timing of DRG reform.

 Table S3 presents the sensitivity analyses by redefining the NMB threshold using the 25th percentile and the mean. The results for AMI patients remained largely consistent with those of the main model after the threshold adjustment, whereas the NMB for cancer patients was not statistically significant when the mean was used as the threshold.

 The pre-trend assessment showed no evidence that the COVID-19 period was significantly associated with changes in the pre-reform trajectories of the main VBH indicators, as shown in Table S4. In the models further adjusted for the COVID-19 period, the estimated post-reform level and slope changes were broadly consistent with the baseline ITS results, as shown in Table S5. These findings suggest that the hospital-level estimates were generally robust to potential COVID-19-related disturbances in the pre-intervention period.

###  Impact of DRG Reform on Hospitalization Costs and Clinical Outcome at the Individual-Level

 The individual-level results in Table 3 show markedly different impact patterns of the DRG payment reform on AMI patients and cancer patients. For AMI patients, the changes were mainly manifested as a slope change rather than an immediate level change, suggesting that the reform impact is gradually emerging. In terms of costs, no significant level change was observed; however, the slope decreased significantly (Coeff. = -0.012, 95% CI: -0.016 to -0.007), indicating a sustained downward trend in hospitalization costs following DRG implementation. For LHS, neither the level change nor the trend change reached statistical significance. In terms of clinical outcomes, mortality showed no significant immediate change, but its slope increased slightly and reached statistical significance (Coeff. = 0.126, 95% CI: 0.029 to 0.223). Neither the level nor the trend change for successful outcomes was significant, and the readmission rate also showed no significant level or trend change. Overall, the impact of DRG reform among AMI patients is reflected in the continuous downward trend of costs, while its impact on hospital stay duration and quality outcomes was limited.

**Table 3:** Patient-Level Changes in Hospitalization Costs and Clinical Outcomes After Diagnosis-Related Group Reform Using Generalized Linear Model and Logistic Regression

**Characteristics**	**Change**	**AMI (N = 16564)**		**Cancers (N = 74356)**	
		**Coeff.**	**95% CI**	**Coeff.**	**95% CI**
Total cost	Level change	0.006	-0.025~0.038	-0.109***	-0.138~0.081
	Slope change	-0.010***	-0.014~-0.006	0.022***	0.018~0.026
LHS	Level change	-0.010	-0.065~0.045	-0.0001	-0.029~0.028
	Slope change	0.007	-0.002~0.013	0.024***	0.020~0.028
Mortality	Level change	0.246	-0.483~0.976	0.336	-0.450~1.121
	Slope change	0.134**	0.039~0.228	0.059	-0.045~0.163
Successful outcome	Level change	0.295	-0.066~0.655	0.639***	0.524~0.754
	Slope change	-0.031	-0.083~0.021	0.008	-0.008~0.024
30-day readmission	Level change	0.188	-0.311~0.686	-0.301***	-0.453~-0.150
	Slope change	-0.008	-0.077~0.061	0.022	-0.001~0.045

Abbreviations: AMI, acute myocardial infarction; CI, confidence interval; LHS, length of hospital stay; Coeff., coefficient. Note: * *P* < .05, ** *P* < .01, *** *P* < .001.

 In contrast, the changes among cancer patients were mainly characterized by level changes, manifesting as immediate structural adjustments after the implementation. Total cost decreased significantly immediately after implementation (Coeff. = -0.108, 95% CI: -0.137 to -0.079); however, the slope subsequently increased (Slope change: Coeff. = 0.021, 95% CI: 0.017 to 0.025), suggesting a gradual upward trend in costs over subsequent periods. There was no significant level change in the LHS, but the slope change increased significantly (Coeff. = 0.028, 95% CI: 0.024 to 0.032). Mortality showed no significant level or trend change. Successful outcomes improved significantly immediately after the reform (Coeff. = 0.632, 95% CI: 0.516 to 0.748), without significant slope change, indicating that the improvement was immediate and subsequently sustained. The readmission rate showed a significant immediate decrease (Coeff. = -0.251, 95% CI: -0.404 to -0.097), and the slope change was not significant. Overall, DRG reform was associated with an immediate decrease in costs and improvement in some outcomes among cancer patients, but costs and hospital stay lengths showed a certain upward trend in the later period, while the mortality did not change significantly.

 The pre-reform assessment showed that the COVID-19 period was significantly associated mainly with selected outcomes among cancer patients, including total cost, LHS, and readmission, as shown inTable S6. No significant association between the COVID-19 period and patient-level outcomes was observed among AMI patients. This suggests that pandemic-related disturbances may have primarily affected pre-reform patient-level outcomes among cancer patients. However, after further adjustment for the COVID-19 period in the patient-level models, the main post-reform estimates remained broadly consistent with the baseline results, as shown inTable S7. These findings suggest that, although COVID-19 may have affected some cancer patient-level outcomes, the main patient-level results were generally robust to adjustment for the COVID-19 period.

###  Impact of DRG Reform on Hospital-Level Value-Based Performance: Concurrent Policy Controls and Ordinary Least Squares With Newey–West Standard Errors

 To reduce potential interference from concurrent policies, this study re-estimated the model by including variables for lockdown measures, MSPA, and CDPP. Results are presented in Table 4. The overall results show that the estimated pattern of post-reform changes on the hospital-level value performance of AMI and cancer patients remains consistent after controlling for concurrent policies. For AMI patients, after controlling for the three concurrent policies, SPC did not observe significant changes in level changes, but the slope changes remained significantly increasing (Lockdown: Coeff. = 0.001, 95% CI: 0.0004 to 0.002; MSPA: Coeff. = 0.002, 95% CI: 0.001 to 0.002; CDPP: Coeff. = 0.001, 95% CI: 0.001 to 0.002). Similarly, CPS exhibited no significant immediate level changes but demonstrated a significant downward slope (Lockdown: Coeff. = -0.164, 95% CI: -0.259 to -0.069; MSPA: Coeff. = -0.255, 95% CI: -0.324 to -0.186; CDPP: Coeff. = -0.217, 95% CI: -0.308 to -0.128). Correspondingly, NMB did not show significant level changes, but its slope changes were significantly increased (Lockdown: Coeff. = 42.888, 95% CI: 3.580 to 82.195; MSPA: Coeff. = 73.516, 95% CI: 34.376 to 112.656; CDPP: Coeff. = 57.066, 95% CI: 19.434 to 94.698).

**Table 4:** Adjusted Hospital-Level Changes in Value-Based Healthcare Indicators After Diagnosis-Related Group Reform: Concurrent Policy Controls

**Characteristics**	**Change**	**Lockdown**				**MSPA**				**CDPP**			
		**AMI (N = 60)**		**Cancers (N = 60)**		**AMI (N = 60)**		**Cancers (N = 60)**		**AMI (N = 60)**		**Cancers (N = 60)**	
		**Coeff.**	**95% CI**	**Coeff.**	**95% CI**	**Coeff.**	**95% CI**	**Coeff.**	**95% CI**	**Coeff.**	**95% CI**	**Coeff.**	**95% CI**
SPC	Level change	-0.002	-0.006~0.002	0.090***	0.062~0.120	-0.001	-0.003~0.001	0.103***	0.069~0.137	-0.005	-0.010~0.0004	0.100***	0.070~0.131
	Slope change	0.001**	0.0004~0.002	-0.006	-0.031~0.020	0.002***	0.001~0.002	-0.016	-0.042~0.011	0.001***	0.001~0.002	-0.011	-0.036~0.013
CPS	Level change	0.330	-0.417~1.078	-0.331**	-0.511~0.152	0.186	-0.193~0.564	-0.376***	-0.575~0.176	0.804	-0.100~1.708	-0.349***	-0.517~-0.182
	Slope change	-0.164**	-0.259~-0.069	0.016	-0.148~0.179	-0.255***	-0.324~-0.186	0.057	-0.00~0.213	-0.217***	-0.308~0.128	0.042	-0.126~0.211
NMB	Level change	-83.055	-312.514~146.405	346.605	-5.495~698.704	-52.284	-179.772~75.205	401.710*	8.160~795.259	-195.020	-547.181~157.142	422.036*	18.479~825.592
	Slope change	42.888*	3.580~82.195	11.817	-157.010~180.644	73.516***	34.376~112.656	-48.888	-229.379~131.603	57.066**	19.434~94.698	-6.921	-160.320~146.477

Abbreviations: AMI, acute myocardial infarction; CI, confidence interval; Coeff., coefficient; SPC, success per cost; CPS, Cost per success; NMB, net monetary benefit; MSPA, medical service price adjustment; CDPP, centralized drug procurement program. Note: (1) * *P* < .05, ** *P* < .01, *** *P* < .001; (2) Models were estimated using Prais–Winsten regression.

 For cancer patients, after controlling the same policies, SPC showed a significant positive level change (Lockdown: Coeff. = 0.090, 95% CI: 0.062 to 0.120; MSPA: Coeff. = 0.103, 95% CI: 0.069 to 0.137; CDPP: Coeff. = 0.100, 95% CI: 0.070 to 0.131). CPS showed significant negative level changes (Lockdown: Coeff. = -0.331, 95% CI: -0.511 to -0.152; MSPA: Coeff. = -0.376, 95% CI: -0.575 to -0.176; CDPP: Coeff. = -0.349, 95% CI: -0.517 to -0.182). NMB also showed a significant positive level change (MSPA: Coeff. = 401.710, 95% CI: 8.160 to 795.259; CDPP: Coeff. = 422.036, 95% CI: 18.479 to 825.592). The slope changes of each indicator did not reach statistical significance, suggesting that the reform effect for cancer patients mainly manifested as an immediate level improvement. In summary, after separately controlling for lockdown measures, MSPA, and CDPP, AMI patients showed a value improvement pattern dominated by slope changes, while cancer patients showed a value improvement pattern dominated by an immediate level jump.

 After conducting robustness tests using ordinary least squares with Newey–West standard errors, the results of the AMI remained largely unchanged, with the significance and direction of both level and slope effects consistent with the main model, supporting the consistency of the estimates. For the cancer group, however, after applying Newey–West standard errors, the slope changes also reached statistical significance; however, the magnitude of the level-change coefficients remained substantially larger than those of the slope changes, indicating that the reform effect was still driven primarily by immediate level shifts, with relatively modest long-term trend effects. Overall, the robustness analyses did not alter the main conclusions. Table S8 shows the results after using ordinary least squares with Newey–West standard errors.

###  Impact of DRG Reform on Patient-Level Costs and Clinical Outcomes: Robustness Analyses With Concurrent Policy Controls, Month-of-Year Fixed Effects, Calendar-Month Clustering, and Mixed-Effects Models

 To evaluate the robustness of the individual-level results after controlling for different concurrent policies, this study re-estimated the models by including variables for lockdown measures, MSPA, and CDPP. The results showed that the post-reform change patterns on AMI and cancer patients remained broadly consistent. For AMI patients, no significant immediate change in the total cost was observed, but the slope showed a significant decrease across all three models (Lockdown: Coeff. = -0.012, 95% CI: -0.016 to -0.007; MSPA: Coeff. = -0.015, 95% CI: -0.020 to -0.010; CDPP: Coeff. = -0.015, 95% CI: -0.019 to -0.011), suggesting that the cost continued to decrease over time. The slope change for mortality also showed a significant increase in all three models (Lockdown: Coeff. = 0.126, 95% CI: 0.029 to 0.223; MSPA: Coeff. = 0.143, 95% CI: 0.025 to 0.261; CDPP: Coeff. = 0.115, 95% CI: 0.019 to 0.211). No statistically significant changes were observed for the other indicators.

 For cancer patients, the total cost showed a significant immediate decrease in all three models (Lockdown: Coeff. = -0.108, 95% CI: -0.137 to -0.079; MSPA: Coeff. = -0.055, 95% CI: -0.093 to -0.018; CDPP: Coeff. = -0.119, 95% CI: -0.147 to -0.090), while the slope changes all showed significant increases (Lockdown: Coeff. = 0.021, 95% CI: 0.017 to 0.025; MSPA: Coeff. = 0.027, 95% CI: 0.022 to 0.032; CDPP: Coeff. = 0.017, 95% CI: 0.012 to 0.021). The slope change of LHS also showed a significant increase (Lockdown: Coeff. = 0.028, 95% CI: 0.024 to 0.032; MSPA: Coeff. = 0.033, 95% CI: 0.023 to 0.042; CDPP: Coeff. = 0.017, 95% CI: 0.013 to 0.021). The successful outcome showed a significant immediate improvement in all three models (Lockdown: Coeff. = 0.632, 95% CI: 0.516 to 0.748; MSPA: Coeff. = 0.623, 95% CI: 0.470 to 0.776; CDPP: Coeff. = 0.629, 95% CI: 0.513 to 0.745). The readmission rate showed a significant immediate decrease in all three models (Lockdown: Coeff. = -0.251, 95% CI: -0.404 to -0.097; MSPA: Coeff. = -0.283, 95% CI: -0.487 to -0.080; CDPP: Coeff. = -0.226, 95% CI: -0.381 to -0.071) (Table 5). No statistically significant changes were observed in the remaining slope change. Overall, after separately controlling for lockdown measures, MSPA, and CDPP, AMI patients showed a continuous decrease in costs over time but an increasing trend in mortality rate. Meanwhile, cancer patients exhibited an immediate decrease in costs, accompanied by an improvement in successful outcomes and a reduction in readmission rates.

**Table 5:** Patient-Level Changes in Costs and Clinical Outcomes After Diagnosis-Related Group Reform With Adjustment for Concurrent Policies

**Characteristics**	**Change**	**Lockdown**				**MSPA**				**CDPP**			
		**AMI (N = 16564)**		**Cancers (N = 74356)**		**AMI (N = 16564)**		**Cancers (N = 74356)**		**AMI (N = 16564)**		**Cancers (N = 74356)**	
		**Coeff.**	**95% CI**	**Coeff.**	**95% CI**	**Coeff.**	**95% CI**	**Coeff.**	**95% CI**	**Coeff.**	**95% CI**	**Coeff.**	**95% CI**
Total cost	Level change	0.012	-0.020~0.044	-0.108***	-0.137~-0.079	-0.033	-0.071~0.004	-0.055**	-0.093~-0.018	0.022	-0.009~0.054	-0.119***	-0.147~-0.090
	Slope change	-0.012***	-0.016~-0.007	0.021***	0.017~0.025	-0.015***	-0.020~-0.010	0.027***	0.022~0.032	-0.015***	-0.019~-0.011	0.017***	0.012~0.021
LHS	Level change	-0.009	-0.064~0.047	-0.007	-0.035~0.022	-0.047	-0.114~0.021	0.083	-0.033~0.199	-0.002	-0.057~0.053	-0.012	-0.040~0.016
	Slope change	0.006	-0.001~0.013	0.028***	0.024~0.032	0.002	-0.007~0.011	0.033***	0.023~0.042	0.004	-0.002~0.011	0.017***	0.013~0.021
Mortality	Level change	0.286	-0.439~1.012	0.172	-0.594~0.939	0.331	-0.575~1.238	0.586	-0.308~1.480	0.389	-0.377~1.156	0.355	-0.444~1.154
	Slope change	0.126*	0.029~0.223	0.099	-0.005~0.203	0.143*	0.025~0.261	0.090	-0.035~0.214	0.115*	0.019~0.211	0.044	-0.062~0.150
Successful outcome	Level change	0.228	-0.143~0.599	0.632***	0.516~0.748	0.276	-0.170~0.721	0.623***	0.470~0.776	0.260	-0.105~0.624	0.629***	0.513~0.745
	Slope change	-0.015	-0.070~0.040	0.012	-0.004~0.029	-0.034	-0.100~0.033	0.006	-0.013~0.026	-0.020	-0.0773~0.032	0.004	-0.012~0.021
30-day readmission	Level change	0.191	-0.299~0.680	-0.251**	-0.404~-0.097	0.387	-0.159~0.933	-0.283**	-0.487~-0.080	0.215	-0.288~0.719	-0.226**	-0.381~-0.071
	Slope change	-0.009	-0.078~0.060	0.009	-0.014~0.033	0.025	-0.061~0.111	0.024	-0.003~0.052	-0.015	-0.085~0.055	-0.017	-0.040~0.006

Abbreviations: AMI, acute myocardial infarction; CI, confidence interval; Coeff., coefficient; MSPA, medical service price adjustment; CDPP, centralized drug procurement program; LHS, length of hospital stay. Note: (1) * *P* < .05, ** *P* < .01, *** *P* < .001; (2) Models were estimated using GLM for continuous outcomes and logistic regression for binary outcomes.

 Additional robustness analyses using standard errors clustered at the year–month level and mixed-effects GLMs yielded consistent coefficient directions and statistical significance compared with the main models, which indicates that the research conclusion has good robustness against different error structure settings and model forms, and the identification of changes does not rely on a specific estimation method. Table S9 shows the results after applying the models using standard errors clustered at the year–month level and mixed-effects GLMs. After adjustment for month-of-year fixed effects and clustering standard errors by calendar month, the main cost-related findings remained broadly consistent with the baseline models, as shown in Table S10. However, patient-level clinical outcomes showed less consistent improvement. In particular, mortality-related estimates became more pronounced, while the previously observed reduction in cancer readmission was attenuated and no longer statistically significant.

###  Subgroups Analysis

 Tables S11 and S12 present the results of an exploratory subgroup analysis of hospital-level value performance based on gender and age stratification. Overall, post-reform changes among cancer patients were consistent across different subgroups with the results of the full patients, being primarily characterized by significant level changes after implementation. In contrast, for AMI patients the reform effects were mainly reflected in slope changes.

 In the gender subgroups, the SPC for both male and female AMI patients showed significant slope changes without significant level changes. CPS showed significant trend decreases in both males and females, but the level changes were not significant. NMB showed significant upward trends but no significant level changes. For cancer patients, regardless of gender, the SPC and CPS showed significant level changes without significant slope changes, consistent with the full patients and suggesting that the impact is mainly an immediate level change.

 In the age subgroups, the changes of AMI patients became more pronounced with increasing age. For individuals under 45 years old, no significant changes were observed in all indicators; for those aged 45–60, only NMB showed a significant upward trend, while the other indicators were not significant; for patients 60 years old and above, SPC, CPS, and NMB all showed significant slope changes, but the level changes did not reach statistical significance. In contrast, cancer patients showed significant level changes in SPC and CPS in all age groups, but the slope changes were not significant, indicating that age stratification did not alter their change patterns.

 Tables S13 and S14 provide the exploratory subgroup analysis results of individual-level hospitalization costs and clinical outcomes. Overall, post-reform changes in hospitalization costs for cancer patients was relatively consistent across different gender and age groups, mainly manifested as a significant decrease in both level change and slope change; while for AMI patients, the impact was only significantly reflected in slope changes, and was more obvious in the elderly population.

 In the gender subgroups, the total hospitalization costs for AMI patients showed a significant downward trend in both men and women, and the levels did not reach statistical significance; the LHS showed a significant slope change only in men, and no significant change was observed in women; mortality also showed a significant slope change only in men, while the other indicators did not show significant differences by gender. In contrast, cancer patients experienced significant immediate reductions in hospitalization costs, improved rates of successful discharge, and lower readmission rates in both sexes; slope effects were significant for some outcomes only among women.

 In the age subgroups, the observed post-reform changes among AMI patients appeared more pronounced with age. No significant changes were observed in all indicators for the population under 45 years old; only mortality and successful discharge outcome showed significant changes for the population aged 45-60; for patients over 60 years old, there was a significant trend of decreasing hospitalization costs, and the mortality rate also showed a significant slope change, while other indicators did not reach statistical significance. In contrast, cancer patients demonstrated a significant level decrease in hospitalization costs across all age groups, accompanied by a significant trend increase in hospital stay days; successful outcomes showed significant improvement across all age groups, and the readmission rate showed a significant level decrease in the middle-aged and elderly groups.

## Discussion

###  Summary and Discussion of Principal Findings

 This study collected data on hospitalized patients with AMI and cancer in a large hospital in western China, providing real-world evidence at the hospital-level and individual-level that China’s DRG reform was associated with VBH-indicator changes. The study compared the different impacts of DRG reform on AMI and cancer hospitalized patients and analysed the differences in the impact of DRG reform on patients of different genders and ages. We found that DRG reform was associated with VBH performance of both AMI and cancer inpatients, while the effects varied across disease types on temporal patterns. Our findings address a knowledge gap in evaluating DRG reform from a VBH perspective and exploring whether clinical heterogeneity shapes reform-related changes. We also explored whether demographic heterogeneity would affect the effect of the DRG reform.

 Our findings suggest that DRG reform was associated with changes in selected hospital-level VBH indicators. The constructed indicators, SPC, CPS, and NMB, changed in directions consistent with improved cost-outcome efficiency for both AMI and cancer inpatients. However, these findings should not be interpreted as evidence of comprehensive improvement in VBH, because the hospital-level indicators were exploratory and some patient-level clinical outcomes did not improve consistently. At the individual-level, DRG reform was associated with reduced total costs, which is consistent with earlier studies.^28,29^ In contrast, a significantly positive slope change in mortality was observed among AMI patients, and length of stay increased over time among cancer patients. These findings highlight potential quality risks that may accompany cost containment under DRG payment, consistent with previous studies.^30,31^

 This study observed an improving trend in the VBH indicators at the hospital-level, while some outcomes showed adverse changes at the individual-level. Such cross-level inconsistency may stem from multiple factors. First, indicators at hospital-level are derived from aggregated, risk-adjusted data and reflect average performance, whereas individual-level analyses evaluate patient-specific outcomes; the two approaches differ in statistical scale and indicator composition.^32^ Even after risk adjustment, aggregated measures may still mask individual heterogeneity.^33^ Therefore, even if the overall performance of the institution improves, there may still be cases where some patients’ outcomes do not improve simultaneously in a certain indicator. Furthermore, the VBH indicators are a composite measurement of multiple dimensions such as cost, efficiency, and quality. The overall gains of these indicators may be mainly driven by cost control, while individual quality dimensions may not improve simultaneously.^34^ The additional patient-level models with month-of-year fixed effects and calendar-month clustered standard errors further supported this cross-level inconsistency. These results suggest that cost-related changes after DRG reform were relatively robust, but improvements in hospital-level VBH indicators may have been driven more by changes in cost efficiency than by consistent improvements in individual-level clinical outcomes.

###  Contrasting Level and Slope Changes in Acute Myocardial Infarction and Cancer

 Our study suggested differences in post-reform changes in value-based, cost, and clinical indicators between AMI and cancer inpatients. Specifically, changes among AMI inpatients were primarily observed as slope changes, whereas changes among cancer patients were mainly observed as level changes. One possible explanation is the differing clinical and cost structure of these conditions. AMI is acute, time-sensitive, and clinically uncertain; delays in diagnosis or treatment can have serious consequences.^35,36^ Physicians may therefore be more conservative when treating possible AMI because of diagnostic uncertainty and perceived risk.^37,38^ These features may limit the immediate cost-compression space after DRG reform and make post-reform changes more gradual.

 The upward slope in AMI mortality should be interpreted cautiously, but it warrants policy attention. Several plausible mechanisms may explain this pattern. First, AMI patients often require rapid, high-intensity rescue care, and fixed payment incentives may create pressure to control costs unless adequate risk adjustment and protection mechanisms are in place.^39,40^ Second, if payment reform is not paired with process-of-care monitoring, administrative data cannot rule out changes in treatment intensity, timeliness, or access to advanced procedures among high-risk patients.^15^ These mechanisms were not directly tested in this study, but they suggest that DRG payment for acute care conditions should be accompanied by targeted quality monitoring, including mortality, timeliness of treatment, readmissions, and outcomes among clinically complex AMI patients.

 Compared to AMI treatment, there are already relatively mature clinical pathways in cancer treatment, with less uncertainty and greater predictability in treatment. Studies have shown that the implementation of standard treatment pathways can significantly reduce medical costs in cancer treatment.^41,42^ Based on these treatment pathways, DRG payment sets fixed payment standards for the same disease group, allowing hospitals to quickly implement cost control based on existing treatment pathways in the early stages of policy implementation, resulting in timely cost savings, manifested as level changes.

###  Exploratory Assessment of Subgroup Differences

 Through exploratory subgroup analysis based on gender and age, the observed post-reform changes showed some differences among AMI inpatients across age groups. The changes appeared more pronounced in the group older than 60 years, while no consistent changes were observed in those younger than 60 years. In contrast, no clear pattern was found when stratified by gender. For cancer inpatients, no clear pattern differences were observed after stratification by gender and age. Because no formal interaction tests were conducted, these subgroup results should be interpreted as exploratory. They may reflect greater cost and clinical complexity among older AMI patients, but they should not be interpreted as confirmatory evidence that older adults benefited more from DRG reform 41-43. Future studies with richer clinical variables and formal interaction testing are needed to validate these patterns.

###  Implications for Policy-Makers

 The results of this study have implications for improving the DRG payment system, but these implications should be interpreted within the limits of the study design. First, DRG payment should not be assumed to show uniform associations across different disease types. A review report by the Belgian Health Care Knowledge Centre found that DRG payment has limitations for high-risk and high-uncertainty diseases, and that payment approaches beyond DRG alone may be needed 45. Second, payment reform should be closely integrated with clinical treatment pathways to improve operability in practice. Third, protection mechanisms for high-risk patients should be strengthened to reduce potential implicit inequities. Fourth, for acute care conditions such as AMI, DRG implementation should be paired with targeted quality monitoring of mortality, timeliness of care, readmissions, and outcomes among clinically complex patients. Finally, policy evaluation should track both hospital-level cost-outcome efficiency and individual-level patient outcomes.

###  Limitations

 Although this study evaluated the association between DRG reform and the realization of VBH goals among AMI and cancer inpatients using real-world hospitalization data, several limitations need to be acknowledged. First, this study was a single-center ITS analysis without a concurrent control group. Data from a single hospital limited our ability to separate the possible influence of the DRG reform from other time-varying factors. Although we considered concurrent policies, secular time trends, and the full COVID-19 period, the influence of unobserved time-varying factors could not be completely ruled out. The additional pre-trend and sensitivity analyses suggested that the main findings were unlikely to be driven solely by COVID-19-related disturbances, but residual pandemic-related confounding may still exist. Therefore, the results should be interpreted as temporal associations rather than definitive causal effects. Future studies could include appropriate control groups to strengthen causal inference.

 Second, the analysis was limited by the administrative data available from the HIS and medical-record homepage database. Although this study included demographic, socioeconomic, and hospitalization-related variables as much as possible for risk adjustment, important clinical severity indicators, such as AMI severity, treatment timeliness, cancer stage, biomarkers, and long-term outcomes, were unavailable. The study also relied on administrative coding and could not independently adjudicate every diagnosis, procedure, or outcome. These data limitations may have affected the measurement of both clinical outcomes and risk adjustment, and future studies with richer clinical data could further improve the robustness of the findings.

 Third, there is still room for refinement of the VBH indicators constructed in this study. In particular, NMB was included as an exploratory hospital-level value indicator. Because there is no unified willingness-to-pay threshold for one successful inpatient outcome in China, we used the median cost per successful treatment in the pre-reform period to define the NMB threshold. This approach allowed us to construct a value measure within the available data, but it may also introduce potential circularity because the threshold was derived from the same pre-reform cost and outcome structure used in the study. In the sensitivity analyses, when the mean was applied as the threshold, the NMB result for cancer patients was no longer statistically significant, suggesting that NMB was sensitive to threshold specification. Therefore, the NMB results should be interpreted cautiously and regarded as supplementary to SPC, CPS, and the patient-level cost and clinical outcome indicators. Future research could incorporate external willingness-to-pay thresholds and additional outcome measures, such as long-term survival or patient-reported outcomes, to develop a more comprehensive VBH evaluation framework.

 Fourth, some outcome and subgroup results should be interpreted with caution. The mortality outcome had a low event rate, particularly in subgroup analyses, so statistically significant mortality-related findings should be regarded as signals requiring further monitoring rather than definitive evidence of harm. In addition, no formal multiple-testing adjustment or interaction testing was conducted. Therefore, subgroup analyses and secondary outcome analyses should be considered exploratory rather than confirmatory.

 Finally, this study found heterogeneity by age and gender among AMI patients, whereas no significant subgroup differences were observed among cancer inpatients. However, the administrative inpatient data did not permit direct examination of the underlying mechanisms. Therefore, the explanations for disease-specific change patterns were based on clinical pathway characteristics and prior literature. Empirical evidence on the interaction between disease type, demographic characteristics, and payment reform also remains limited, which constrains external validation of the proposed mechanisms. Future studies incorporating richer data sources, mixed-method approaches, and hospitals from different regions of China would help to further examine how disease characteristics and demographic differences influence the effects of payment system reforms.

## Conclusion

 This study found that DRG reform was associated with changes in selected hospital-level VBH indicators, while individual-level clinical outcomes did not show consistent improvement. The observed changes were mainly characterized by slope changes among AMI patients and level changes among cancer patients. Exploratory subgroup analyses suggested possible age-related differences among AMI patients, but these findings require validation through formal interaction testing and richer clinical variables. The results suggest that post-reform changes may vary across diseases with different clinical pathways, cost structures, and risk profiles. During the promotion of DRG reform, disease-specific payment strategies should be combined with stronger risk adjustment and targeted monitoring of patient-level outcomes, especially for acute and high-risk conditions. Therefore, this study provides preliminary evidence on how DRG reform may be associated with hospital-level efficiency and patient-level outcomes in one tertiary hospital in western China.

## Acknowledgments

 We thank the National Natural Science Foundation of China for financial support.

## Disclosure of artificial intelligence (AI) use

 ChatGPT was used only for language editing, spelling correction, and grammar checking throughout the manuscript.

## Ethical issues

 Ethical approval for this study was granted under protocol IRB#2023-TYSQ-10-306.

## Conflicts of interest

 Authors declare that they have no conflicts of interest.

## Supplementary files


Supplementary file 1 contains Tables S1-S14.
