# Supplementary material for: Association Between China’s DRG Reform and Value-Based Healthcare Orientation: Evidence From Inpatients With Acute Myocardial Infarction and Cancer in Tertiary Hospitals in Western China
Source: Int J Health Policy Manag. 2026 Jul 14;15:9669. doi: 10.34172/ijhpm.9669 (PMC13770236; doi:10.34172/ijhpm.9669)
Supplement: Supplementary file 1 [file ijhpm-15-9669-s001.pdf]

Article title: Association Between China's DRG Reform and ValueBased Healthcare Orientation: Evidence From Inpatients With Acute Myocardial Infarction and Cancer in Tertiary Hospitals in Western China

Journal name: International Journal of Health Policy and Management (IJHPM)

Authors' information: Haoran Li[1,2], Fukun Zhu[1], Tao Xie[3], Ning Zhang[4], Austen El-Osta[2], Zhonghan Wang[1], Ying Mao[1]*

[1]School of Public Policy and Administration, Xi'an Jiaotong University, Xi'an, China.

[2]School of Public Health, Imperial College London, London, UK.

[3]School of Political Science and Public Administration, Wuhan University, Wuhan, China.

[4]Vanke School of Public Health, Tsinghua University, Beijing, China.

***Correspondence to:** Ying Mao; Email: mao_ying@mail.xjtu.edu.cn

Citation: Li H, Zhu F, Xie T, et al. Association between China's DRG reform and value-based healthcare orientation: evidence from inpatients with acute myocardial infarction and cancer in tertiary hospitals in western China. Int J Health Policy Manag. 2026;15:9669. doi:10.34172/ijhpm.9669

Supplementary file 1

Table S1. Durbin–Watson statistics before and after Prais–Winsten correction for autocorrelation.

Characteristics	Durbin–Watson	AMI	Cancers
SPC	Before	0.778	0.308
	After	2.379	1.909
CPS	Before	0.757	0.360
	After	2.378	2.102
NMB	Before	0.737	0.564
	After	2.160	2.006

Note: Durbin–Watson (DW) statistics close to 2 indicate no first-order autocorrelation; values substantially below 2 suggest positive autocorrelation, and values above 2 suggest negative autocorrelation. DW values before Prais–Winsten regression were <1 across outcomes, indicating strong positive autocorrelation. After applying Prais–Winsten AR(1) correction, DW values were approximately 2, suggesting that residual autocorrelation was adequately addressed.

Table S2. Adjusted Changes in hospital level Value-Based Performance Associated with DRG Reform using OLS with Newey–West standard errors.

Characteristics	Change	AMI (N=60)		Cancers (N=60)	
		Coef.	95%CI	Coef.	95%CI
SPC	Level Change	-0.003	-0.008~0.002	0.156*	0.031~0.281
	Slope Change	0.001***	-0.0008~0.001	-0.011***	-0.015~-0.006
CPS	Level Change	0.426	-0.427~1.278	-1.222*	-2.310~-0.134
	Slope Change	-0.177***	-0.233~-0.121	0.069***	0.033~0.104
NMB	Level Change	-208.067	-445.212~29.078	932.108*	152.837~1711.378
	Slope Change	58.583***	40.545~76.622	-37.240*	-68.721~-5.760

Notes: 1) *P<0.05, **P<0.01, ***P<0.001. Coef., coefficient.

Table S3. Adjusted Changes in hospital level Value-Based Performance Associated with DRG Reform by Gender Subgroups using Prais–Winsten regression.

Characteristics	Change	Male				Female			
		AMI (N=60)		Cancers (N=60)		AMI (N=60)		Cancers (N=60)	
		Coef.	95%CI	Coef.	95%CI	Coef.	95%CI	Coef.	95%CI
SPC	Level Change	-0.002	-0.007~0.003	0.075***	0.048~0.101	-0.007	-0.016~0.001	0.112***	0.071~0.153
	Slope Change	0.001***	0.0005~0.002	-0.006	-0.025~0.013	0.001***	0.001~0.002	-0.015	-0.044~0.013
CPS	Level Change	0.285	-0.560~1.160	-0.467**	-0.768~-0.167	1.376	-0.286~3.038	-0.326**	-0.531~-0.122
	Slope Change	-0.159**	-0.248~-0.069	0.017	-0.145~0.179	-0.282**	-0.436~0.127	0.034	-0.094~0.162
NMB	Level Change	-73.367	-286.148~139.414	209.393*	13.871~4004.915	-45.631	-145.178~53.915	195.274	-8.633~399.182
	Slope Change	35.577*	3.948~67.207	4.427	-68.602~77.456	10.843*	0.603~21.083	-13.169	-98.896~72.558

Notes: 1) *P<0.05, **P<0.01, ***P<0.001. Coef., coefficient

Table S4. Adjusted Changes in hospital level Value-Based Performance Associated with DRG Reform by Age Groups using Prais–Winsten regression.

Characteristics	Change	<45				45-60				>60			
		AMI (N=60)		Cancers (N=60)		AMI (N=60)		Cancers (N=60)		AMI (N=60)		Cancers (N=60)	
		**Coef.**	**95%CI**	**Coef.**	**95%CI**	**Coef.**	**95%CI**	**Coef.**	**95%CI**	**Coef.**	**95%CI**	**Coef.**	**95%CI**
**SPC**	Level Change	-0.001	-0.012~0.010	0.068*	0.016~0.121	0.005	-0.013~0.023	0.156***	0.123~0.192	-0.004	-0.010~0.002	0.082***	0.048~0.11
	Slope Change	0.001	-0.0001~0.002	-0.011	-0.043~0.022	0.0003	-0.002~0.002	-0.012	-0.034~0.010	0.001***	0.001~0.002	-0.009	-0.032~0.01
**CPS**	Level Change	-0.349	-3.326~2.629	-0.413*	-0.781~0.046	-0.537	-3.131~2.057	-0.483***	-0.696~-0.271	0.775	-0.368~1.917	-0.469**	-0.785~-0.1
	Slope Change	-0.238	-0.519~0.043	0.025	-0.076~0.126	-0.066	-0.356~0.223	0.016	-0.108~0.139	-0.220***	-0.328~-0.111	0.037	-0.104~0.17
**NMB**	Level Change	-4.979	-36.183~26.224	47.117	-39.842~134.076	-37.982	-261.254~185.291	193.948**	79.798~308.097	-93.210	-308.822~122.402	216.996	-13.287~447.
	Slope Change	2.567	-2.360~7.495	-3.121	-26.439~20.197	16.232	-11.229~43.692	-3.095	-45.360~39.170	31.472**	8.465~54.478	-5.590	-99.969~88.7

Notes: 1) *P<0.05, **P<0.01, ***P<0.001. Coef., coefficient

Table S5. Impact of DRG Reform on Individual-Level Value-Based Performance: Results Clustered Standard Errors, and Mixed-Effects GLM.

Characteristics	Change	Clustered SEs (year–month)				Mixed-effects generalized linear model			
		AMI (N=16,564)		Cancers (N=74,356)		AMI (N=16,564)		Cancers (N=74,356)	
		Coef.	95%CI	Coef.	95%CI	Coef.	95%CI	Coef.	95%CI
Total cost	Level Change	0.006	-0.055~0.067	-0.109**	-0.188~-0.031	0.007	-0.025~0.038	-0.038**	-0.061~-0.015
	Slope Change	-0.010***	-0.015~-0.005	0.022***	0.012~0.032	-0.010***	-0.014~-0.006	0.005**	0.002~0.008
LHS	Level Change	-0.010	-0.071~0.051	-0.0002	-0.127~0.126	-0.008	-0.053~0.036	0.066***	0.047~0.085
	Slope Change	0.007	-0.002~0.015	0.024***	0.012~0.036	0.010**	0.004~0.016	0.004**	0.001~0.006
Mortality	Level Change	0.246	-0.322~0.815	0.336	-0.590~1.262	0.281	-0.471~1.034	0.336	-0.424~1.096
	Slope Change	0.134**	0.066~0.202	0.059	-0.040~0.158	0.137**	0.044~0.230	0.059	-0.039~0.157
Successful Outcome	Level Change	0.295	-0.080~0.669	0.639**	0.221~1.058	0.301	-0.065~0.667	0.867***	0.687~1.046
	Slope Change	-0.031	-0.072~0.010	0.008	-0.037~0.053	-0.032	-0.083~0.020	0.015	-0.010~0.040
Readmission	Level Change	0.188	-0.319~0.694	-0.301	-0.789~0.186	0.201	-0.319~0.721	-0.240**	-0.401~-0.079
	Slope Change	-0.008	-0.090~0.074	0.022	-0.010~0.054	-0.008	-0.077~0.062	0.022	-0.002~0.046

Notes: 1) *P<0.05, **P<0.01, ***P<0.001. Coef., coefficient.

Table S6. Changes in Hospitalization Costs and Clinical Outcome using Gender Subgroups GLM and Logistic Regression.

**Characteristics**	**Change**	**Male**				**Female**			
		**AMI (N=16,564)**		**Cancers (N=74,356)**		**AMI (N=16,564)**		**Cancers (N=74,356)**	
		**Coef.**	**95%CI**	**Coef.**	**95%CI**	**Coef.**	**95%CI**	**Coef.**	**95%CI**
**Total cost**	Level Change	-0.008	-0.042~0.027	-0.117***	-0.162~-0.070	0.068	-0.011~0.148	-0.104***	-0.140~0.068
	Slope Change	-0.009***	-0.014~-0.004	0.020***	0.013~0.027	-0.016**	-0.025~-0.007	0.023***	0.019~0.028
**LHS**	Level Change	-0.032	-0.093~0.029	-0.036	-0.077~0.005	0.092	-0.032~0.216	0.035	-0.004~0.074
	Slope Change	0.008*	0.001~0.016	0.023***	0.017~0.028	-0.001	-0.015~0.014	0.025***	0.020~0.030
**Mortality**	Level Change	0.190	-0.643~1.023	0.635	-0.292~1.562	0.466	-1.002~1.933	-0.412	-1.853~1.030
	Slope Change	0.168**	0.066~0.270	0.026	-0.102~0.154	0.031	-0.188~0.250	0.138	-0.036~0.312
**Successful Outcome**	Level Change	0.400	-0.030~0.830	0.055***	0.0418~0.679	-0.009	-0.685~0.667	0.950***	0.695~1.206
	Slope Change	-0.033	-0.096~0.031	0.031**	0.012~0.049	-0.023	-0.114~0.068	-0.058**	-0.092~-0.024
**Readmission**	Level Change	0.345	-0.207~0.896	-0.207*	-0.414~-0.001	-0.521	-1.664~0.622	-0.409***	-0.632~-0.187
	Slope Change	-0.019	-0.097~0.060	-0.002	-0.034~0.029	0.027	-0.110~0.165	0.045**	0.012~0.079

Notes: 1) *P<0.05, **P<0.01, ***P<0.001. Coef., coefficient.

Table S7. Changes in Hospitalization Costs and Clinical Outcome using Age Subgroups GLM and Logistic Regression.

**Characteristics**	**Change**	**<45**				**45-60**				**>60**			
		**AMI (N=16,564)**		**Cancers (N=74,356)**		**AMI (N=16,564)**		**Cancers (N=74,356)**		**AMI (N=16,564)**		**Cancers (N=74,356)**	
		**Coef.**	**95%CI**	**Coef.**	**95%CI**	**Coef.**	**95%CI**	**Coef.**	**95%CI**	**Coef.**	**95%CI**	**Coef.**	**95%CI**
**Total cost**	Level Change	0.018	-0.084~0.120	-0.190***	-0.264~-0.115	0.004	-0.048~0.057	-0.128***	-0.171~-0.085	0.010	-0.032~0.052	-0.073**	-0.117~0.030
	Slope Change	-0.014	-0.030~0.002	0.027***	0.017~0.036	-0.001*	-0.016~0.002	0.022***	0.016~0.028	-0.011***	-0.016~-0.005	0.020***	0.014~0.027
**LHS**	Level Change	-0.149	-0.330~0.032	-0.036	-0.110~0.039	-0.062	-0.150~0.025	-0.013	-0.060~0.035	0.033	-0.040~0.106	0.016	-0.023~0.056
	Slope Change	0.033**	0.009~0.056	0.029***	0.020~0.038	0.015*	0.003~0.027	0.025***	0.019~0.032	-0.0003	-0.009~0.008	0.022***	0.016~0.027
**Mortality**	Level Change	1.193	-1.904~4.291	0.772	-2.925~4.470	0.692	-1.690~3.074	0.285	-1.527~2.097	0.097	-0.719~0.913	0.250	-0.655~1.155

	Slope Change	0.142	-0.302~0.585	0.012	-0.292~0.317	0.265*	0.013~0.518	0.053	-0.171~0.276	0.133*	0.027~0.239	0.067	-0.054~0.188
**Successful Outcome**	Level Change	0.755	-1.106~2.615	1.131***	0.586~1.677	1.110*	0.043~2.177	0.641***	0.405~0.876	0.147	-0.252~0.547	0.596***	0.459~0.733
	Slope Change	-0.067	-0.347~0.213	-0.070	-0.141~0.001	-0.133	-0.279~0.014	-0.011	-0.042~0.021	-0.016	-0.073~0.041	0.023*	0.003~0.042
**Readmission**	Level Change	0.959	-1.109~3.028	-0.329	-0.766~0.107	-0.262	-1.429~0.904	-0.271*	-0.053~-0.013	0.251	-0.342~0.845	-0.355**	-0.563~0.148
	Slope Change	-0.014	-0.220~0.193	-0.002	-0.071~0.067	0.036	-0.135~0.207	0.021	-0.017~0.060	-0.022	-0.103~0.060	0.028	-0.003~0.059

Notes: 1) *P<0.05, **P<0.01, ***P<0.001. Coef., coefficient.

Table S8. Level and slope changes in mean and 25th percentile NMB for AMI and cancers

Characteristics	Change	AMI (N=60)		Cancers (N=60)	
		Coef.	95%CI	Coef.	95%CI
NMB_Mean	Level Change	-80.578	-303.346~142.191	-13.477	-441.290~414.336
	Slope Change	42.106*	3.325~80.888	-21.533	-381.438~338.372
NMB_25	Level Change	-80.727	-351.60~190.216	437.653*	48.338~826.969
	Slope Change	42.982*	4.757~81.209	-10.6360	-166.219~144.947

Notes: 1) *P<0.05, **P<0.01, ***P<0.001. Coef., coefficient.

Table S9. Model fit statistics and multicollinearity diagnostics for risk-adjustment and individual-level regression models

Characteristics		AMI	Cancers
R^2	SPC	0.673	0.221
	CPS	0.667	0.387
	NMB	0.073	0.124
VIF		1.05	

Table S10. Association Between the COVID-19 Period and Pre-Reform Hospital-Level VBH Indicators

Characteristics	AMI (N=48)		Cancers (N=48)	
	Coef.	95%CI	Coef.	95%CI
SPC	-0.001	-0.005~0.002	0.01	-0.002~0.02
CPS	0.26	-0.40~0.92	-0.17	-0.42~0.07
NMB	-0.55	-153.12~152.02	437.38	132.90~741.87

Notes: 1) *P<0.05, **P<0.01, ***P<0.001. Coef., coefficient. 2) Coefficients represent the estimated association of the COVID-19 period indicator with each hospital-level VBH indicator during the pre-reform period, after adjusting for the underlying linear time trend.

Table S11. Association Between the COVID-19 Period and Pre-Reform Individual-Level VBH Indicators

Characteristics	AMI (N=12,289)		Cancers (N=42,770)	
	**Coef.**	**95%CI**	**Coef.**	**95%CI**
**Total cost**	0.01	-0.02~0.04	0.19***	0.16~0.23
**LHS**	-0.04	-0.09~0.01	0.07***	0.03~0.11
**Mortality**	-0.28	-1.30~0.74	0.10	-0.98~1.18
**Successful Outcome**	0.11	-0.36~0.58	-0.17	-0.39~0.04
**Readmission**	0.34	-0.24~0.91	0.87***	0.69~1.05

Notes: 1) *P<0.05, **P<0.01, ***P<0.001. Coef., coefficient. 2) Coefficients represent the estimated association of the COVID-19 period indicator with each institution-level indicators during the pre-reform period, after adjusting for the underlying linear time trend.

Table S12. Hospital-Level Changes in VBH Indicators After DRG Reform With Adjustment for the Full COVID-19 Period

Characteristics	Change	AMI (N=60)		Cancers (N=60)	
		**Coef.**	**95%CI**	**Coef.**	**95%CI**
**SPC**	Level Change	-0.002	-0.006~0.002	0.10***	0.07~0.13
	Slope Change	0.001**	0.0003~0.002	-0.01	-0.04~0.01
**CPS**	Level Change	0.29	-0.40~0.98	-0.37***	-0.53~-0.18
	Slope Change	-0.15**	-0.25~-0.05	0.03	-0.13~0.20
**NMB**	Level Change	-80.26	-313.78~153.27	394.02*	52.01~736.03
	Slope Change	42.28*	2.70~91.86	1.12	-176.64~178.88

Notes: 1) *P<0.05, **P<0.01, ***P<0.001. Coef., coefficient.

Table S13. Individual-Level Changes in VBH Indicators After DRG Reform With Adjustment for the Full COVID-19 Period

Characteristics	Change	AMI (N=16,564)		Cancers (N=74,356)	
		Coef.	95%CI	Coef.	95%CI
Total cost	Level Change	0.02	-0.01~0.06	-0.11***	-0.14~-0.08
	Slope Change	-0.01***	-0.01~-0.001	0.03***	0.02~0.03
LHS	Level Change	-0.03	-0.1~0.03	-0.02	-0.05~0.01
	Slope Change	0.01	-0.002~0.01	0.02***	0.02~0.03
Mortality	Level Change	0.06	-0.75~0.88	0.29	-0.58~1.16
	Slope Change	0.13*	0.03~0.22	0.06	-0.05~0.17
Successful Outcome	Level Change	0.34	-0.06~0.75	0.62***	0.49~0.74
	Slope Change	-0.03	-0.08~0.03	0.005	-0.01~0.02
Readmission	Level Change	0.25	-0.30~0.82	-0.20*	-0.37~-0.03
	Slope Change	0.01	-0.07~0.07	0.05***	0.02~0.07

Notes: 1) *P<0.05, **P<0.01, ***P<0.001. Coef., coefficient.

Table S14. Patient-Level Changes in VBH Indicators After DRG Reform With Adjustment for Month-of-Year Fixed Effects and Calendar-Month Clustered Standard Errors

Characteristics	Change	AMI (N=16,564)		Cancers (N=74,356)	
		Coef.	95%CI	Coef.	95%CI
**Total cost**	Level Change	0.03	-0.03~0.09	-0.20***	-0.29~-0.11
	Slope Change	-0.01***	-0.02~-0.01	0.03***	0.02~0.04
**LHS**	Level Change	0.03	-0.02~0.08	-0.05	-0.16~0.07
	Slope Change	0.001	-0.006~0.009	0.03***	0.02~0.04
**Mortality**	Level Change	0.67*	0.002~1.34	0.10	-0.52~0.72
	Slope Change	0.08*	0.004~0.15	0.08*	0.01~0.15
**Successful Outcome**	Level Change	0.35	-0.003~0.70	1.01***	0.58~1.43
	Slope Change	-0.04	-0.08~0.01	-0.03	-0.09~0.02
**30-day Readmission**	Level Change	0.16	-0.38~0.70	-0.44	-1.21~0.32
	Slope Change	-0.004	-0.08~0.07	0.04	-0.04~0.11

Notes: 1) *P<0.05, **P<0.01, ***P<0.001. Coef., coefficient.
